# Simultaneous Estimation of Escitalopram and Clonazepam in Tablet Dosage Forms Using HPLC-DAD Method and Optimization of Chromatographic Conditions by Box-Behnken Design

**DOI:** 10.3390/molecules27134209

**Published:** 2022-06-30

**Authors:** Ahmed I. Foudah, Sultan Alshehri, Faiyaz Shakeel, Mohammed H. Alqarni, Tariq M. Aljarba, Prawez Alam

**Affiliations:** 1 Department of Pharmacognosy, College of Pharmacy, Prince Sattam Bin Abdulaziz University, Al-Kharj 11942, Saudi Arabia; a.foudah@psau.edu.sa (A.I.F.); m.alqarni@psau.edu.sa (M.H.A.); t.aljarba@psau.edu.sa (T.M.A.); 2 Department of Pharmaceutical Sciences, College of Pharmacy, AlMaarefa University, Ad Diriyah 13713, Saudi Arabia; sshehri@mcst.edu.sa; 3 Department of Pharmaceutics, College of Pharmacy, King Saud University, Riyadh 11451, Saudi Arabia; faiyazs@fastmail.fm

**Keywords:** Box-Behnken design, RP-HPLC, ICH guidelines, stability studies, assay

## Abstract

The study aimed to develop a new reverse-phase high-performance liquid chromatography (RP-HPLC) method with diode array detection (DAD) detection for simultaneous estimation of escitalopram (EST) and clonazepam (CZP) in tablet dosage forms with a quality by design (QbD) approach. The chromatographic conditions were optimized by Box-Behnken design (BBD) and developed method was validated for the linearity, system suitability, accuracy, precision, robustness, sensitivity, and solution stability according to International Council for Harmonization (ICH) guidelines. EST and CZP standard drugs peaks were separated at retention times of 2.668 and 5.046 min by C-18 column with dimension of 4.6 × 100 mm length and particle size packing 2.5 µm. The mobile phase was methanol: 0.1% orthophosphoric acid (OPA) (25:75, *v*/*v*), with a flow rate of 0.7 mL/min at temperature of 26 °C. The sample volume injected was 20 µL and peaks were detected at 239 nm. Using the standard calibration curve, the % assay of marketed tablet was founded 98.89 and 98.76 for EST and CZP, respectively. The proposed RP-HPLC method was able to detect EST and CZP in the presence of their degradation products, indicating the stability-indicating property of the developed RP-HPLC method. The validation parameter’s results in terms of linearity, system suitability, accuracy, precision, robustness, sensitivity, and solution stability were in an acceptable range as per the ICH guidelines. The newly developed RP-HPLC method with QbD application is simple, accurate, time-saving, and economic.

## 1. Introduction

The combination of escitalopram (EST) and clonazepam (CZP) is used for the treatment of anxiety disorder. EST is an antidepressant and CZP as an anticonvulsant, muscle relaxant, and anxiolytic agent. EST is a pure s-enantiomer of the racemic, bicyclic phthalates derivatives citalopram, belonging to class selective serotonin reuptake inhibitor have shown potent pharmacological effects [1,2]. Few pieces of literature are available for the simultaneous estimation of EST and CZP in dosages form based on spectrometric, colorimetric, and chromatographic analysis. An ultraviolet (UV) spectrophotometric method has been reported for the simultaneous estimation of EST and CZP in tablet formulations, which was simple, accurate, and precise [3]. An UV spectrophotometric method has also been used for the determination of CZP in combination with paroxetine hydrochloride in combined tablet dosage forms, which was also simple, accurate, and precise [4]. Zero order spectrophotometry method was also used for the estimation of EST alone in tablet formulations [5]. An UV spectrophotometry method was used to estimate CZP alone in tablet dosage forms [6]. Synchronous fluorescence spectroscopy method has been used for enantiometric assay of EST and to estimate its in-process impurities [7]. All spectrometry-based assays were simple, accurate, and precise [3,4,5,6,7]. However, the reported spectrometry-based methods were not sensitive enough for the determination of EST or CZP, either alone or in combined dosage forms compared to developed HPLC method. 

Various high-performance liquid chromatography (HPLC) methods have also been reported for the determination of EST or CZP, either alone or in combined dosage forms [8,9,10,11,12]. A stability-indicating HPLC method was used for the simultaneous estimation of EST and CZP in bulk drug and tablet formulations [8]. Some HPLC methods have also been used to estimate CZP alone in tablet formulations [9,10]. HPLC method has also been reported to estimate EST in combination with flupentixol hydrochloride in tablet dosage forms [11]. Estimation of EST in combination and combined dosage form has been reported by several researchers in different dosage forms or biological fluids by liquid chromatography (LC) coupled with mass spectrometer (MS) [12]. Reported HPLC methods for the determination of EST or CZP were linear, accurate, precise, and robust [9,10,11,12]. However, a single HPLC method was stability-indicating one [8]. In addition, the reported HPLC method for the simultaneous determination of EST and CZP was not rapid and time-saving compared to the developed HPLC method [8]. The statistical optimization of any of the reported analytical methods of EST and CZP determination was not performed in literature. Several LC analytical techniques have shown many disadvantages like time-consuming, expensive, column block due to high buffer concentration, rare availability solvent like tetrabutyl, and the high flow rate [13]. These shortcomings can be minimized by adopting the quality by design (QbD) approach in analysis. 

Researchers have developed simple and robust methods suitable for the estimation of multiple drugs in a single dosage form. Optimization of chromatographic condition with QbD approach requires less time, accuracy, and is cost-effective for qualitative and quantitative analysis. QbD approaches of analysis have been reported in active pharmaceutical ingredients (API) and herbal formulation earlier [14,15,16]. Central composite design (CCD) usually had axial points outside the cube. These points could not be in the region of interest because they are beyond the safe operating limits [17]. Box-Behnken design (BBD) does not have axial points and hence all the design points fall within the safe operating limits [18]. In addition, CCD requires large number of experiments compared to the BBD [17,18]. Therefore, BBD was used to optimize chromatographic conditions instead of CCD. This research work has been designed to estimate the EST and CZP in marketed formulation with BBD approach for the optimization of chromatographic conditions in HPLC method development and validated method as per International Council for Harmonization (ICH) guidelines. It is a new, simple, accurate, and economic method for the simultaneous estimation of EST and CZP. 

## 2. Materials and Methods

### 2.1. Materials

EST and CZP were procured from Sigma Aldrich (St. Louis, MO, USA). HPLC grade methanol was procured from SD Fine Chemicals (Mumbai, India). Ortho-phosphoric acid (OPA) (85–88% purity) was purchased from Loba Chemical (Mumbai, India).

### 2.2. Instrumentation

Chromatographic conditions were developed for the analytical technique using Agilent HPLC system (Agilent-1100, Santa Clara, CA, USA), Gradient System, diode array detection (DAD) detector, and Software (Chemstation). The column was Zorbax RP C-18 (Santa Clara, CA, USA) with dimension 4.6 × 100 mm length and particle size packing 2.5 µm. The precolumn was not used in this study.

### 2.3. Optimization of Chromatographic Condition with QbD Concept

Analytical target profile (ATP): The objective here is to optimize the chromatographic conditions to improve the quality of the peak in simultaneous estimation, the peaks must be highly resolved with good tailing factor. The quality specification of the analytical method should be achieved by ATP [19,20]. 

### 2.4. Risk Assessment 

**Figure 1:**
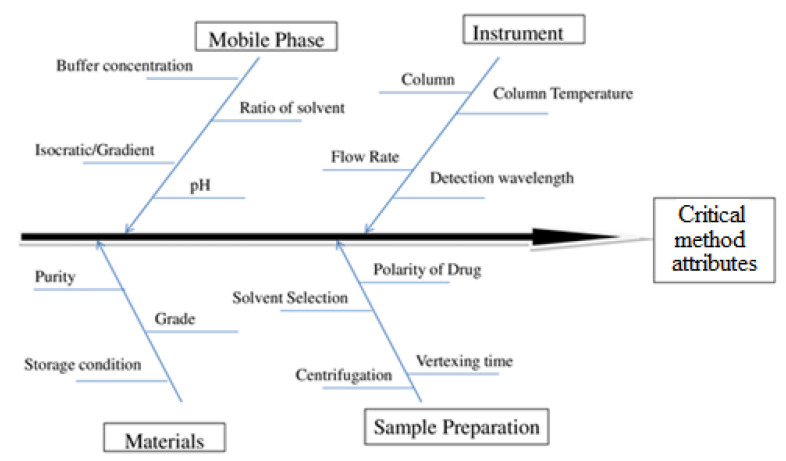
Factor affecting the critical method attributes.

In HPLC method development, many factors influence the quality of separation like column configuration, mobile phase, flow rate, detection wavelength, column temperature, and injection volume, which affects the performance of the instrument. Among the numerous factors identified the critical method attributes (CAA) for this constructed the fishbone diagram and was carried systematic risk analysis shown in Figure 1 [21]. 

### 2.5. Optimization of Chromatographic Conditions Using BBD

Chromatographic conditions were optimized using BBD (Design Expert 13.0.3.0 software Stat-Ease Inc., Minneapolis, MN, USA). The total of seventeen trials have been taken and out of seventeen, five trials were optimized. Others trials differed from each other because of the effects of its interaction over the factors. Here, optimization was performed in simultaneous drugs, considering three factors that affect the retention time and tailing factor response of each drug [22,23]. 

### 2.6. Method Development

The mobile phase was optimized for methanol: 0.1% OPA (25:75, *v*/*v*) and having a flow rate of 0.7 mL/min at a temperature of 26 °C. The sample volume used was 20 µL and the DAD detection was performed at 239 nm. The total run time was about 8 min in the system, the retention time of EST and CZP was 2.668 and 5.046 min and tailing factors values were 0.76 and 0.66 for EST and CZP, respectively.

### 2.7. Preparation of Standard Solutions

The standard solution was prepared in the methanol. The weighted accurately 100 mg of EST and 5 mg of CZP and then dissolved in 100 mL of methanol in a volumetric flask. This is the first stock solution and its concentration was 1000 µg/mL for EST and 50 µg/mL for CZP [24,25].

### 2.8. Determination of λ_max_

The samples were scanned at a concentration of drug 10 µg/mL of EST and 0.5 µg/mL of CZP in the wavelength range of 200–400 nm and the λ_max_ was determined at 239 nm. 

### 2.9. Preparation of Calibration Curve

Five dilutions were prepared from the stock solution over the concentration range (10 µg/mL for EST and 0.5 µg/mL for CZP), (20 µg/mL for EST and 1 µg/mL for CZP), (30 µg/mL for EST and 1.5 µg/mL for CZP), (40 µg/mL for EST and 2 µg/mL for CZP), and (50 µg/mL for EST and 2.5 µg/mL for CZP). The linearity is the ability to show the response with concentration based on Beers-Lambert law. Also, the limit of detection (LOD) and limit of quantification (LOQ) values were calculated.

### 2.10. Method Validation

The newly developed HPLC method was validated according to the ICH guidelines. Experiments were performed and developed method was validated for various validation parameters, such as system suitability, linearity, precision, accuracy, robustness, LOD, LOQ, and solution stability [26,27]. For the system suitability test, the variations for two parameters, retention time and tailing factor were recorded in terms of the percentage of coefficient of variance (%CV) [28]. For the determination of linearity, the average peak areas were plotted against the concentrations (*n* = 3) and then linearity was evaluated using the calibration curve to calculate a determination coefficient (R^2^), slope, and intercept [29]. Precision was reported in terms of inter-day and intraday variations by considering the three quality control samples of each in low-quality control (LQC), middle-quality control (MQC), and high-quality control (HQC) level. The precision results were expressed in terms of %CV. The traditional method was used for the determination of accuracy by spiking method [30,31]. The robustness of developed HPLC method determined by introducing some intentional changes in flow rate, mobile phase composition, and detection wavelength [32,33]. The LOD and LOQ values were determined using standard deviation technique reported in literature [26]. The solution stability was determined at MQC of both drugs (30 μg/mL EST) and (1.5 μg/mL CZP) at a temperature of 25 °C for 14 days and 2–8 °C for 30 days [34,35]. The detailed procedures for all validation parameters are included in Supplementary Materials.

### 2.11. Assay of the Tablet

Twenty tablets (containing both EST and CZP) were weighted and crushed, the total weight of the powder was 0.310 g, the average weight of the tablet was 0.1550 g equivalent to 15.5 mg of EST and CZP. Accurately weighed 0.155 g (containing 15.5 mg of EST and CZP) was transferred in a volumetric flask and the volume was adjusted to 100 mL using methanol. The prepared mixture was sonicated for 30 min and filtered with 0.45 µm membrane filter. Further, a series of dilutions was prepared with mobile phase over the range of developed calibration curves. The obtained solutions were injected six times to the system and the % assay was calculated from the calibration curve.

### 2.12. Forced Degradation Study

Standard mixtures of both drugs were exposed in different conditions and performed the chromatographic analysis of degraded products [36].

#### 2.12.1. Acid Hydrolysis

Accurately weighted 100 mg of standard EST and CZP were taken and transferred into three sets of 250 mL round bottom flask and then 20 mL 1N HCl was added to all flasks and refluxed on the heated mantle for 45 min at 80 °C.

#### 2.12.2. Alkali Hydrolysis

In 1N NaOH, accurately weighed 100 mg standard EST and CZP were transferred into a round bottom flask and refluxed on the heated mantel at 80° C for 60 min.

#### 2.12.3. Oxidative Degradation

In three 250 flasks, accurately weighed 100 mg standard EST and CZP were transferred and then 20 mL of 6% H_2_O_2_ was added to all flasks and refluxed on the heated mantle at 80 °C for 2 h.

#### 2.12.4. Thermal Degradation

The accurately weighted 100 mg of standard EST and CZP was transferred into Petri-dish and spread it with a spatula, then placed the petri-dish in the hot air oven for 1 h at 80 °C. The heated samples were taken into a 100 mL volumetric flask and dissolved in diluents to make up the volume up to the mark. Approximately 1 mL of the sample was taken and transferred into a 10 mL volumetric flask and diluted it to a 10 mL in a volumetric flask, filtered through 0.45 μm Millipore nylon filter, and chromatographic analysis was performed. 

## 3. Results and Discussion

### 3.1. Optimization 

The results of BBD application, after performing the risk assessment the factors selected are, % methanol in the mobile phase, flow rate of mobile phase, and detection wavelength. The response of drugs EST and CZP are reported as retention time and tailing factor. The trial of samples is presented in Table 1. Three independent variables such as the %methanol in mobile phase (A), flow rate (B), and λ_max_ (C) were studied. Four measured responses (dependent variables) were retention time of EST (response 1), tailing factor of EST (response 2), retention time of CZP (response 3), and tailing factor of CZP (response 4). The retention time of EST was observed in the range of 2.505–2.779 min. The retention time of five center points of BBD for EST was found to be 2.668 min. The tailing factor of EST was observed in the range of 0.71–0.79. The tailing factor of five center points of BBD for EST was found to be 0.76. The retention time of CZP was observed in the range of 4.975–5.069 min. The retention time of five center points of BBD for CZP was found to be 5.046 min. The tailing factor of CZP was observed in the range of 0.61–0.69. The tailing factor of five center points of BBD for CZP was found to be 0.66. 

#### 3.1.1. The Retention Time of EST

EST in the optimized condition is presented in Table 1. Out of seventeen trials, the five trials have been optimized (13–17). The inbuilt one-way analysis of variance (ANOVA) for the quadratic model is significant. Model summary statistics value is shown in Table 2. The *p* value for most of the model terms is less than 0.0500, which indicates that the entire model is significant. 

**Figure 2:**
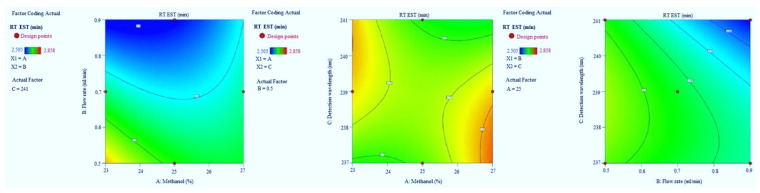
Contour plots for escitalopram (EST) retention time in terms of AB, AC, and BC.

**Figure 3:**
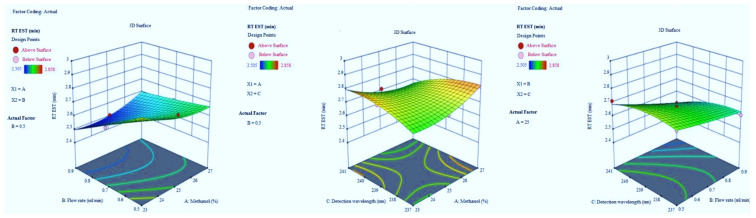
3D responses for EST retention time in terms of AB, AC, and BC.

Adequate precision measures the signal-to-noise ratio. The adequate precision of greater than 4 is desirable [14]. The recorded adequate precision value of 21.766 indicates an adequate signal (Table 2). The R^2^ value was predicted to be 0.9786, which indicated that the 97.86% of variable was explained by the model and only 2.14% was as a result of chance. This model can be used to navigate the design space. Final equation in terms of coded factors A (+0.0592), B (−0.0668), C (−0.0338), AB (+0.0575), AC (−0.0650), BC (−0.0260), A2 (+0.0455), B2 (+0.0045) and C2 (−0.0385). Model graphs of this in terms of contour plots (AB, AC, and BC) are shown in Figure 2 and 3D responses (AB, AC, and BC) are presented in Figure 3.

#### 3.1.2. Tailing Factor of EST 

**Figure 4:**
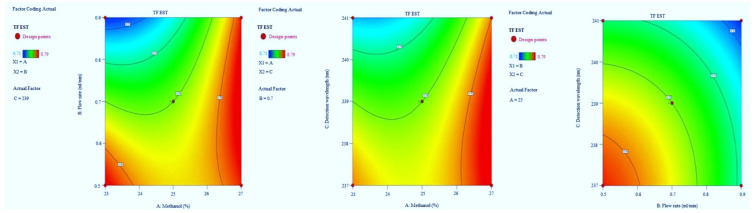
Contour plots for EST tailing factor in terms of AB, AC, and BC.

**Figure 5:**
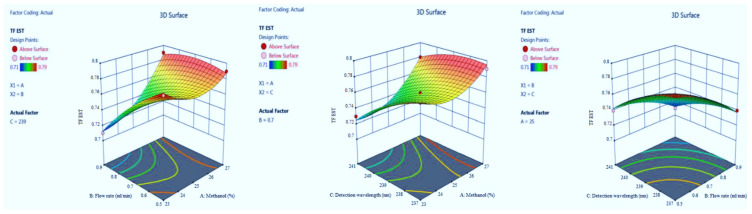
3D responses for EST tailing factor in terms of AB, AC, and BC.

The peak symmetry confirmed by the value of tailing factor, here finalized it by BBD application with inbuilt ANOVA for the quadratic model. The optimized trial presented in Table 1 and model summary statistics in Table 2. The F-value of the model implies 92.37 which indicates it is significant. The model terms *p*-values are less than 0.0500. The R^2^ value was predicted to be 0.9809, which indicated that the 98.09% of variable was explained by the model and only 1.91% was as a result of chance. Model graphs of this response in terms of contour plots (AB, AC, and BC) are shown in Figure 4 and 3D responses (AB, AC, and BC) in Figure 5. The final equation in terms of coded factors A (+0.0175), B (−0.0200), C (−0.0175), AB (+0.0200), AC (+0.0100), BC (+0.0050), A2 (+0.0175), B2 (−0.0075) and C2 (−0.0075).

#### 3.1.3. The Retention Time of CZP

**Figure 6:**
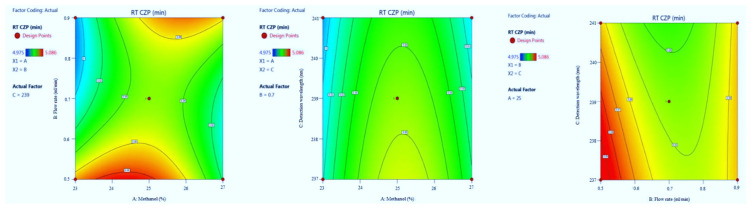
Contour plots for clonazepam (CZP) retention time in terms of AB, AC, and BC.

**Figure 7:**
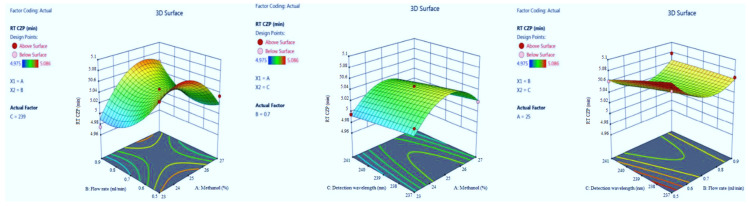
3D responses for CZP retention time in terms of AB, AC, and BC.

CZP in the optimized condition is presented in Table 1. Model summary statistics value is presented in Table 2. The value of adequate precision is 10.756, indicating an adequate signal. The R^2^ value was predicted to be 0.9238, which indicated that the 92.38% of variable was explained by the model and 7.62% was as a result of chance. Final equation in terms of coded factors A (+0.0054), B (−0.0084), C (−0.0090), AB (+0.0305), AC (−0.0008), BC (+0088), A2 (−0.0390), B2 (+0.0280) and C2 (−0.0007). Model graphs in terms of contour plots (AB, AC, and BC) are shown in Figure 6 and 3D responses (AB, AC, and BC) in Figure 7.

#### 3.1.4. Tailing Factor of CZP 

**Figure 8:**
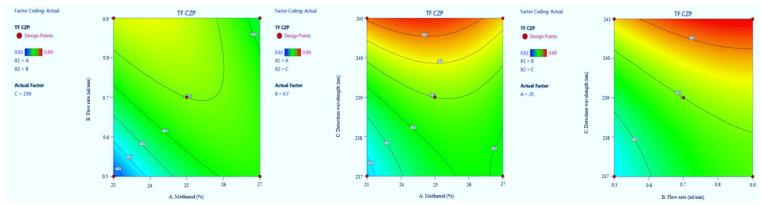
Contour plots for CZP tailing factor time in terms of AB, AC, and BC.

**Figure 9:**
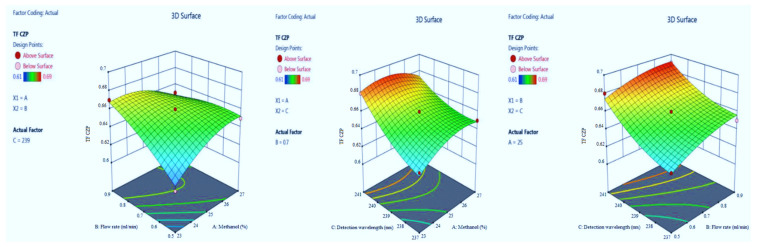
3D responses for CZP tailing factor in terms of AB, AC, and BC.

The F-value of the model implies 27.61 which indicate it is significant. The model terms *p*-values are also less than 0.0500. The R^2^ value was predicted to be 0.9726, which indicated that the 97.26% of variable was explained by the model and only 2.74% was as a result of chance. Model graphs of this response in terms of contour plots (AB, AC, and BC) are shown in Figure 8 and 3D responses (AB, AC, and BC) in Figure 9. The final Equation in terms of coded factors A (+0.0037), B (+0.0112), C (+0.0200), AB (−0.0150), AC (−0.0075), BC (−0.0025), A2 (−0.0100), B2 (−0.0050) and C2 (+0.0075).

### 3.2. Calibration Curve

**Figure 10:**
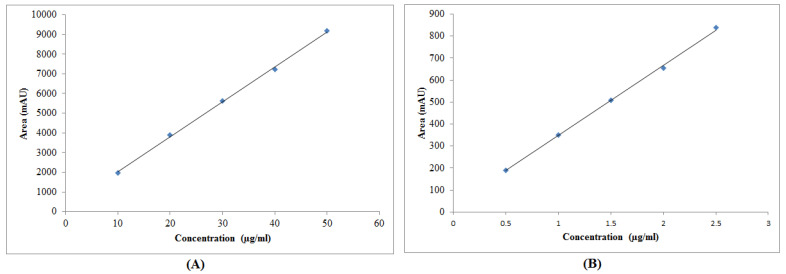
Representative calibration curve of (**A**) EST and (**B**) CZP.

**Figure 11:**
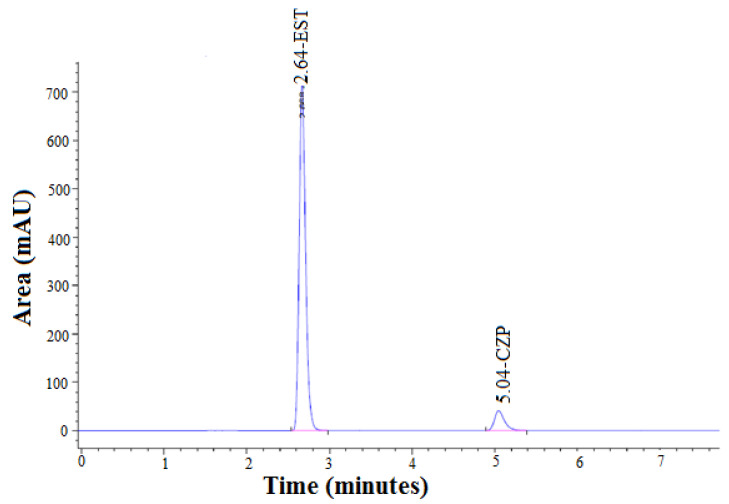
Representative high-performance liquid chromatography (HPLC) chromatograms of standard EST (R_t_ = 2.64 min) and CZP (R_t_ = 5.04 min).

The representative calibration curves for EST and CZP are presented in Figure 10. The regression linearity equation for EST was recorded as Y = 177.82x + 242.49 with R^2^ = 0.999 (Figure 10A). The linearity equation for CZP was recorded as Y = 320.03x + 28.326 with R^2^ = 0.999 (Figure 10B). In equations, Y is the measured area and x is the concentration. The calibration curve for EST was linear in the range of 10–50 µg/mL. However, the calibration curve for CZP was linear in the range of 0.5–2.5 µg/mL. The R^2^ value for both drugs was highly acceptable. The mean of standard deviation and %CV was 12.80 and 0.19%, respectively for EST and 0.39 and 0.09%, respectively for CZP. Chromatogram of the standard drugs in given retention time is shown in Figure 11. These results suggested good linear relation between the measured area and concentration.

### 3.3. Method Validation

#### 3.3.1. System Suitability Test

The result of the system suitability (*n* = 3) in terms of average value, standard deviation (SD), and %CV of the drug EST parameter retention time was founded 2.64 min, 0.04, and 1.13%, respectively, and for tailing factor, 0.75, 0.02 and 3.2%, respectively. For the CZP retention time, these values were 5.04 min, 0.02 and 0.33%, respectively, and for tailing factor, 0.65, 0.02 and 3.2%, respectively. The retention times of EST and CZP using HPLC method have been reported as 4.42 and 6.53 min, respectively [8]. The total run times have been reported as 15 min for the simultaneous determination of EST and CZP using HPLC method [8]. The recorded retention times of EST (2.64 min) and CZP (5.04 min) in this study were lower than the reported ones. In addition, the total run times of 8 min was much lower than reported run times of 15 min. These results indicated that the proposed HPLC method for the simultaneous determination of EST and CZP was rapid and time-saving compared to the reported HPLC method [8]. Overall, optimized tailing factors, optimized vales of retention times, and low values of %CVs indicated that instrument performance was good. As a result, the proposed RP-HPLC method can be considered reliable for the simultaneous determination of EST and CZP.

#### 3.3.2. Linearity

The results of linearity (*n* = 3) were analyzed based on peak area and concentration. The EST was found to be linear in the concentration range of 10–50 μg/mL. The SD of the peak area and %CV for EST were found to be 12.8 and 0.19%, respectively. The CZP was found to be linear in the concentration range of 0.5–2.5 μg/mL. The SD of the peak area and %CV for CZP were determined as 0.39 and 0.09%, respectively. These results suggested the linearity of the proposed RP-HPLC method for the simultaneous determination of EST and CZP. 

#### 3.3.3. Precision and Accuracy

The results of precision in terms of intra-day and inter-day precisions of EST and CZP are presented in Table 3. The precisions for both drugs were expressed in terms of %CV. The %CVs of intra-day precisions for EST at three different QC levels were recorded as 0.05–0.23%. The %CVs of inter-day precisions for EST at three different QC levels were recorded as 0.17–0.64%. The %CVs of intra-day precisions for CZP at three different QC levels were recorded as 0.23–0.32%. The %CVs of inter-day precisions for CZP at three different QC levels were recorded as 0.41–1.19%. The low values of %CVs for both drugs suggested the precision of the proposed RP-HPLC method for the simultaneous determination of EST and CZP. The results of accuracy measurement for both drugs were expressed as the percentage recovery and results are included in Table 3. The predetermined concentration of 10 μg/mL was selected as the target concentration and spiked with extra 80%, 100%, and 120% amount for the accuracy measurement. The %recovery for EST was recorded as 100.39–102.36% with %CVs of 0.26–1.43%. The %recovery for CZP was found to be 100.02–102.24% with %CVs of 0.01–0.44%. The higher values of %recoveries for both drugs indicated the accuracy of the proposed RP-HPLC method for the simultaneous determination of EST and CZP. 

#### 3.3.4. Solution Stability 

The solution stability for EST and CZP was determined in terms of %recovery and %CV at room temperature 25 °C and refrigerator temperature (2–8 °C). The %recovery and %CV of EST (30 μg/mL) was recorded as 98.85% and 0.58%, respectively at 25 °C after 14 days. The %recovery and %CV of CZP (1.5 μg/mL) was found to be 98.75% and 0.68%, respectively at 25 °C after 14 days. The %recovery and %CV of EST at 2–8 °C was determined as 99.65% and 0.78%, respectively. The %recovery and %CV of CZP was recorded as 101.75% and 0.88%, respectively. The high values of %recoveries and low values of %CV suggested the solution stability of both compounds.

#### 3.3.5. Robustness Study

The concentration of EST (40 μg/mL) and CZP (2 μg/mL) was taken for the experiment (*n* = 3). The results of the intentional change in parameters such as flow rate, mobile phase composition, and detection wavelength are presented in Table 4 in terms of mean area, SD and %CV. The %CVs for EST were recorded as 0.03–0.43%. The %CVs for CZP were found to be 0.22–0.61%. The %CV below 2% indicates the robustness of the developed RP-HPLC method for the simultaneous determination of EST and CZP. 

### 3.4. Assay of Tablet

The percentage assay results of the tablet were founded 98.89 and 98.76 of EST and CZP respectively in comparison to the standard of both drugs. The result is in between 90–110% of the label claim. 

### 3.5. Forced Degradation Studies 

**Figure 12:**
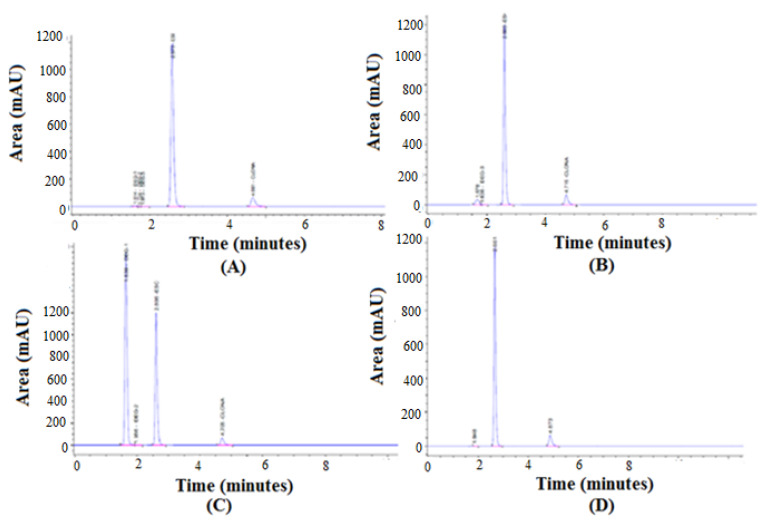
Representative HPLC chromatograms recorded under (**A**) acid, (**B**) base, (**C**) H_2_O_2_, and (**D**) thermal degradation conditions.

The results of the forced degradation studies are presented in Table 5 and chromatograms are presented in Figure 12. The degradation of EST was found to be less than 1% at acid, alkali, oxidative, and thermal degradation conditions. Hence, the EST was highly stable under all degradation conditions. The degradation of CZP was found to be 13.86%, 16.86%, 6.63%, and 0.76% at acid, alkali, oxidative, and thermal degradation conditions, respectively. Hence, the CZP was also sufficiently stable under all degradation conditions. The large peak in Figure 12C was an oxidative degradation peak of CZP [37]. This degradation peak might be a related pharmaceutical impurity of CZP, which is known as CZP related compound A (3-amino-4-2-(chlorohprnyl)-6-nitrocarbostyril) [37,38]. The proposed RP-HPLC method was able to detect EST and CZP simultaneously in the presence of their degradation products, indicating the stability-indicating property. 

## 4. Conclusions

The goal of this study was to develop a simple, accurate, and economic HPLC method for the simultaneous estimation of EST and CZP in marketed formulation. The application of BBD for the simultaneous estimation of EST and CZP is a unique approach, whose advantages are time and cost-saving along with enhancing the quality of analysis by focusing on the quality in process steps. The proposed HPLC method was found to be a simple, economical, accurate, precise, robust, and sensitive for the simultaneous determination of EST and CZP. The proposed HPLC method was able to detect the degradation products of both drugs in the presence of their degradation products, indicating the stability-indicating property of the method. The inbuilt ANOVA results of QbD has shown that it was significant. Both peaks are resolved properly in the chromatogram and the tailing factor value was indicated that the developed peaks are symmetric. Results of the validation parameter were in an acceptable range. Assay results of the marked formulations were within 90–110%. These findings suggested that the proposed HPLC method can be used for the simultaneous determination of EST and CZP in combined dosage forms. 

## Figures and Tables

**Table 1:** Box-Behnken design (BBD) summary.

Run	Factor A (%Metnaol in Mobile Phase)	Factor B (Flow Rate)	Factor C (λ_max_)	Response 1 (Retention Time of EST)	Response 2 (Tailing Factor of EST)	Response 3 (Retention Time of CZP)	Response 4 (Tailing Factor of CZP)
1.	23	0.5	239	2.779	0.79	5.069	0.61
2.	27	0.5	239	2.769	0.79	5.034	0.65
3.	23	0.9	239	2.552	0.71	4.975	0.67
4.	27	0.9	239	2.772	0.79	5.062	0.65
5.	23	0.7	237	2.596	0.78	5.022	0.63
6.	27	0.7	237	2.858	0.79	5.019	0.65
7.	23	0.7	241	2.622	0.73	4.995	0.68
8.	27	0.7	241	2.624	0.78	4.989	0.67
9.	25	0.5	237	2.711	0.79	5.086	0.63
10.	25	0.9	237	2.608	0.74	5.068	0.65
11.	25	0.5	241	2.712	0.74	5.061	0.68
12.	25	0.9	241	2.505	0.71	5.078	0.69
13 *.	25	0.7	239	2.668	0.76	5.046	0.66
14 *.	25	0.7	239	2.668	0.76	5.046	0.66
15 *.	25	0.7	239	2.668	0.76	5.046	0.66
16 *.	25	0.7	239	2.668	0.76	5.046	0.66
17 *.	25	0.7	239	2.668	0.76	5.046	0.66

13–17 * Optimized trial; EST: escitalopram; CZP: clonazipam.

**Table 2:** Model summary statistics.

	EST		CZP	
	Retention Time	Tailing Factor	Retention Time	Tailing Factor
Source	Quadratic	Quadratic	Quadratic	Quadratic
Std.dev.	0.0196	0.0038	0.0132	0.0050
R^2^	0.9786	0.9809	0.9238	0.9726
Adjusted R^2^	0.9510	0.9917	0.8259	0.9374
Predicted R^2^	0.6568	08664	−0.2185	0.5617
Sequential *p*-value	0.0034	<0.0001	0.0012	0.0066
Adequate precision	21.766	14.112	10.756	8.2412

EST: escitalopram; CZP: clonazipam; R^2^: determination coefficient.

**Table 3:** Precision and accuracy data.

	Precision							
	EST				CZP			
QC Sample (μg/mL)	Intraday		Inter-Day		Intraday		Inter-day	
	SD	CV (%)	SD	CV (%)	SD	CV (%)	SD	CV (%)
LOQ	0.96	0.23	5.65	0.64	1.12	0.32	1.43	0.41
MOQ	4.73	0.08	2.76	0.02	1.02	0.23	2.04	0.40
HQC	3.40	0.05	1.47	0.17	1.95	0.29	7.84	1.19
	Accuracy							
Excess drug added (%)	EST				CZP			
	Avg. recovered (%)	CV (%)	SD		Avg. recovered (%)	SD	CV (%)	
80	101.06	1.44	1.43		100.02	0.01	0.01	
100	100.39	0.21	0.21		102.24	0.45	0.44	
120	102.36	0.26	0.26		101.68	0.22	0.22	

EST: escitalopram; CZP: clonazipam; QC: quality control; LQC: low quality control; MQC: middle quality control; HQC: high quality control; SD: standard deviation; percentage of coefficient of variance (%CV).

**Table 4:** Robustness data.

	EST			CZP		
	Mean Area (µAU)	SD	CV (%)	Mean Area (µAU)	SD	CV (%)
Flow rate at 0.6 mL/min	8563.44	2.96	0.03	771.22	1.69	0.22
Flow rate at 0.8 mL/min	6409.23	22.3	0.35	580.10	1.52	0.26
Methanol (24%) + OPA (76%)	7295.60	30.26	0.41	662.60	1.87	0.28
Methanol (26%) + OPA (74%)	7303.25	31.11	0.43	661.12	2.78	0.42
Wavelength 238 nm	7176.40	2.57	0.04	701.40	1.81	0.26
Wavelength 240 nm	7527	9.03	0.12	622.61	3.82	0.61

EST: escitalopram; CZP: clonazipam; SD: standard deviation; percentage of coefficient of variance (%CV); OPA: orthophosphoric acid.

**Table 5:** Results of forced degradation studies.

S. No.	Degradation	Conc. of Standard (µg/mL)	Conc. of Drug Remaining (µg/mL)	Actual Degradation (%)
	EST			
1.	Acid degradation	30.00	29.81	0.63
2.	Basic degradation	30.00	29.97	0.10
3.	H_2_O_2_ degradation	30.00	29.75	0.83
4.	Thermal degradation	30.00	29.81	0.63
	CZP			
1.	Acid degradation	30.00	25.84	13.86
2.	Basic degradation	30.00	24.94	16.86
3.	H_2_O_2_ degradation	30.00	28.01	6.63
4.	Thermal degradation	30.00	29.77	0.76

EST: escitalopram; CZP: clonazipam.

## Data Availability

This study did not report any data.

## Supplementary Materials

The following supporting information can be downloaded at: https://www.mdpi.com/article/10.3390/molecules27134209/s1. The detailed experimental procedures for different validation parameters are included in supplementary materials file.

## Sample Availability

Samples of the compounds EST and CZP are available from the authors.
