# Supplementary material for: Simultaneous Estimation of Escitalopram and Clonazepam in Tablet Dosage Forms Using HPLC-DAD Method and Optimization of Chromatographic Conditions by Box-Behnken Design
Source: Molecules. 2022 Jun 30;27(13):4209. doi: 10.3390/molecules27134209 (PMC9268709; doi:10.3390/molecules27134209)
Supplement: Supplementary file 1 [file molecules-27-04209-s001.zip › molecules-1773228-supplementary.pdf]

Supplementary Materials

Method Validation

System Suitability Test

The performance of the HPLC instrument has been evaluated by performing the system suitability test where the experiment result must be reproducible and for it followed United States Pharmacopoeia (USP) 24/National Formulary (NF) 19. Six batches of the sample were checked and before analysis examine the chromatographic system's reproducibility. The variations for two parameters, retention time and tailing factor were determined in terms of the percentage of coefficient of variation (% CV).

Linearity

The response obtained of the sample solutions to directly proportional to the concentration of analyte is reported as linearity. It was performed by injecting the standard solutions of each drug over the range (n=3). The average peak areas were plotted against the concentrations and then linearity was evaluated using the calibration curve to calculate a coefficient of correlation, slope, and intercept.

Precision and Accuracy

Precision was reported in terms of inter-day and intraday variations, experiments were performed by considering the three quality control samples of each in low-quality control (LQC), middle-quality control (MQC), and high-quality control (HQC) level. For EST, the concentrations of LQC, MQC and HQC were 20 μg/mL, 30 μg/mL, and 40 μg/mL, respectively and for CZP, these were 1 μg/mL, 1.5 μg/mL, and 2 μg/mL, respectively. In each experiment %CV was determined of each level of concentration. The traditional method was followed for the determination of accuracy that is percentage recovery; a pre-quantified concentration of 20 μg/mL of EST sample and for CZP 1 μg/mL were taken then added 0, 50, 100, and 150 % drug solution.

Robustness Study

The developed new HPLC method needs to check its robustness, for this performed a practical with intentional change in the chromatographic condition and examined the result if the variations in the result within the acceptable range as mentioned in guidelines than the developed considered as a robust method. Here, considered the parameters like flow rate, mobile phase composition, and wavelength affect the symmetry of the peak and retention time.

Limit of Detection (LOD) and Limit of quantification (LOQ)

The sensitivity for developed HPLC method was determined as LOD and LOQ using standard deviation method. The blank analyte was injected three replicates (n = 3) and its standard deviation was obtained. The LOD and LOQ for the developed HPLC method were calculated using the following formulae:

$$\mathrm{LOD} = 3.3 \times \frac{\sigma}{S} \quad (1)$$

$$\mathrm{LOQ} = 10 \times \frac{\sigma}{S} \quad (2)$$

where, σ = standard deviation of the blank sample and S = slope of the calibration curve.

Solution Stability

To check the stability of the solution, a quality control sample of level MQC of both drugs (30 μg/mL EST) and (1.5 μg/mL CZP) was prepared and stored at a temperature of 25ºC for 14 days and 2-8 ºC for 30 days and its experimental results reported in terms of % recovery and %CV.
